# A new mechanism for low and temperature-independent elastic modulus

**DOI:** 10.1038/srep11477

**Published:** 2015-06-25

**Authors:** Liangxiang Zhang, Dong Wang, Xiaobing Ren, Yunzhi Wang

**Affiliations:** 1 Center of microstructure science, Multi-Disciplinary Materials Research Center, Frontier Institute of Science and Technology, State Key Laboratory for Mechanical Behavior of Materials, Xi’an Jiaotong University, Xi’an 710049, China; 2 Ferroic Physics Group, National Institute for Materials Science, Tsukuba, 305-0047, Ibaraki, Japan; 3 Department of Materials Science and Engineering, The Ohio State University, 2041 College Road, Columbus, OH 43210, USA

## Abstract

The first Elinvar alloy, FeNiCr, which has invariant elastic modulus over a wide temperature range, was discovered almost 100 years ago by Guillaume. The physical origin of such an anomaly has been attributed to the magnetic phase transition taking place in the system. However, the recent discovery of non-magnetic Elinvar such as multi-functional β-type Ti alloys has imposed a new challenge to the existing theories. In this study we show that random field from stress-carrying defects could suppress the sharp first-order martensitic transformation into a continuous strain glass transition, leading to continued formation and confined growth of nano-domains of martensite in a broad temperature range. Accompanying such a unique transition, there is a gradual softening of the elastic modulus over a wide temperature range, which compensates the normal modulus hardening due to anharmonic atomic vibration, resulting in a low and temperature-independent elastic modulus. The abundance of austenite/martensite interfaces are found responsible for the low elastic modulus.

The elastic modulus of most solids decreases when temperature increases as a consequence of thermal expansion^1^,^2^ and such a temperature dependence of the elastic modulus can be described by the Wachtman’s equation^2^. However, a well-known exception is the Elinvar alloy, Fe_52_Ni_36_Cr_12_^3^, discovered by Guillaume in late 1890s, which has invariant elastic modulus over a wide temperature range. Since then materials of similar anomaly have been reported in ferromagnetic (e.g., FeSiB amorphous alloys^4^) and anti-ferromagnetic (e.g., MnNiCr alloys^5^) systems and they have found many applications in advanced instrumentation that requires high mechanical accuracy such as filter chronometer, seismographs, pressure gauges, mechanical vibrators and delay lines^6^,^7^,^8^. Mechanistic studies^9^,^10^,^11^,^12^ have focused on magnetic phase transitions and the associated magnetoelastic interactions as the possible physical origin of the Elinvar anomaly (Elinvar for short hereafter). For example, the transition between two spin states upon cooling could result in significant elastic modulus softening, which is able to compensate the normal elastic modulus hardening on cooling and leads to Elinvar^11^. However, the recent discovery of non-magnetic Elinvars (e.g., the GUM metals^13^) has imposed a new challenge to the existing theories based on magnetic phase transition.

A gradual modulus softening upon cooling that can compensate normal modulus hardening caused by anharmonic atomic vibration is required for Elinvar. It is well known that martensitic transformations (MTs) are accompanied by elastic modulus softening (acoustic phonon softening)^14^,^15^. However, normal MTs are too sharp and nonlinear due to autocatalysis and under-regulated and rapid growth of domains after nucleation. As a consequence, the modulus softens abruptly within a narrow temperature window around M_s_. However, if the sharp first-order MTs could be smeared out into a continuous transformation over a broad temperature range, then the gradual modulus softening accompanying such a smooth transformation could compensate the normal modulus hardening, offering a new mechanism that could operate in both magnetic and non-magnetic systems. Recently, such a continuous MT has been reported in doped ferroelastic systems^16^,^17^ and is now referred to as strain glass transition (STGT)^18^,^19^ and gradual modulus softening with frequency dispersion has been observed^20^,^21^.

Similar to relaxor ferroelectrics^22^ and ferromagnetic cluster spin glasses^23^ where nanoscale heterogeneities exist in their ferroic order parameters, i.e., polarization and magnetization, respectively, the ferroelastic strain glasses^19^,^24^ are characterized by nanoscale heterogeneities in the transformation strain. Also, all of them have characteristic ferroic dynamics slowing down with decreasing temperature. Note that there are discussions in literature regarding whether the source of the strain disorder (i.e., nanoscale heterogeneities) at lower temperature in a “strain glass” state is due to dynamical arrest of thermally equilibrated strain disorder of higher temperature (“strain liquid”), or quenched-in diffusive disorder (chemical) from still higher temperatures^25^,^26^,^27^, or both. For the Elinvar anomaly that we are concerned in the current study, it makes no difference whether the strain glass state is exactly the analog of liquid melt-quench produced structural glass or any other forms of “amorphization” that have not experienced the typical cooling-induced viscosity-increase liquid-glass transition. The only hypothesis we make is that the random field from point defects changes normal long-range ordered, polytwinned domain structure (“strain crystal”) into nano-domains of individual variants of martensite (“strain glass”), full of strain disorder at interfaces between martensite and retained austenite, and alters the overall characteristics of the martensitic transformation from sharp first-order to continuous. Such an apparently continuous martensitic transformation (i.e., STGT) is accompanied by a gradual softening of the elastic modulus upon cooling that compensates the normal modulus hardening associated with anharmonic atomic vibration, leading to the Elinvar anomaly.

Whether this continuous MT can be used to explain Elinvar and to design new alloys with low and invariant elastic modulus is the focus of the current study. We show by both computer simulation and experiment that STGT in doped ferroelastic systems is indeed accompanied by a continuous modulus softening that leads to Elinvar. In particular, we calculate and later measure the storage (dynamic) and static moduli as function of temperature for normal martensitic systems (low defects concentrations) and strain glass systems (high defects concentrations) and show that the former is accompanied by a sharp elastic modulus softening in a narrow temperature range around the transition temperature, while the latter is accompanied by a gradual modulus softening in a broad temperature range leading to Elinvar. Further analysis of the simulation results shows that the abundance of retained austenite and domain boundaries between austenite and martensite in a strain glass state and their evolution during SGTG are responsible for the observed low and temperature-independent elastic modulus. This study has demonstrated a new and general mechanism for achieving Elinvar in both magnetic and non-magnetic ferroelastic systems.

## Methods

The model system considered in the simulations is a generic single crystal undergoing a proper austenite (A) (cubic) → martensite (M) (tetragonal) transformation in three-dimension (3D). The three deformation variants (also called Bain or correspondence variants) produced by such a MT along the Bain paths are described by three non-conserved order parameter fields, η_1_(**r**), η_2_(**r**) and η_3_(**r**), characterizing their spatial distributions^28^. The stress-free transformation strain (SFTS) experienced by a volume element located at **r** during the transformation is then given by


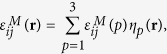
where


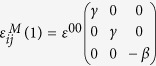

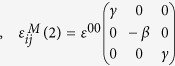

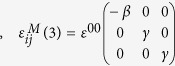
, are the SFTS tensors and *ε*^00^ is the magnitude of the STFS^28^,^29^. In simulations, the following set of model parameters: *γ* = 0.6, *β* = 1.0 and *ε*^00^ = 0.1941), corresponding to the classical Elinvar alloy system, FeNi, have chosen^30^,^31^. Any arbitrary microstructures of the system can then be described by these order parameter fields, e.g., (η_1,_η_2_,η_3_) =  (0,0,0) represents the A phase and (η_1,_η_2_,η_3_) = (1,0,0), (0,1,0) or (0,0,1) represnets one of the three deformation variants of the M phase, respectively.

The local chemical free energy of the system is approximated by a Landau polynomial,


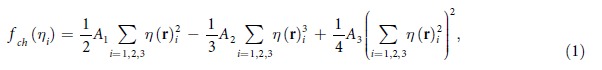


where A_1_, A_2_, A_3_ are the expansion coefficients and 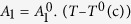, c is a dimensionless average defect concentration, T is temperature, *T*^ 0^ (c) *= T*^ 00^ + *b*·*c* is the critical transition temperture at defect concentration c, with T^00^ being the critical transition temperature at c = 0 and *b* being a constant characterizing the relative strength of the global transition temperature effect (GTTE)^32^ associated with the point defects. The GTTE describes, in an effecitive medium approxiamtion, how the doped defects alter the thermodynamic stability of the M phase relative to the A phase and change the transition temperature of the system.

In addition to their chemical effect, the point defects, in particular their clusters, also generate local lattice distortions^33^. The random stress field associated with the lattice distortions creates the so-called local field effect (LFE)^32^,^34^,^35^. In the simulations, the effect of the local stress field associated with the point defects, 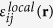, on the Landau potential is described through the following equation^17^,^32^


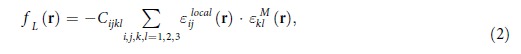


where *C*_ijkl_ is the elastic modulus tensor and 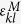 is the SFTS field of martensite defined earlier. Note that the homogeneous modulus assumption for austenite and martensite is used in our simulations.

In gradient thermodynamics of a non-uniform system^36^,^37^,^38^, a non-local gradient energy term, *f*_*gr*_(*η*_*i*_), is introduced to describe contributions from spatial non-uniformity of the order parameters to the total free energy of the system, e.g.,


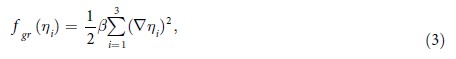


where β is the gradient energy coefficient.

The coherent elastic strain energy, *E*_*el*_, associated with the lattice mismatch between the A and M phases as well as among different variants of the M phase, can be described by Khachaturyan’s microelasticity theory^29^.


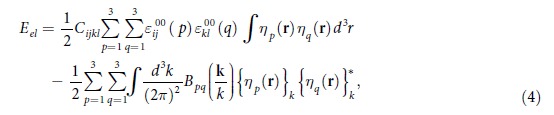


where **k** is the wave vector defined in the reciprocal space, {*η*_*p*_(**r**)}_*k*_ is the Fourier transform of *η*_*p*_(**r**), 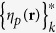 is the complex conjugate of {*η*_*p*_(**r**)}_*k*_, and the kernel 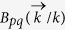 is 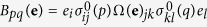, where 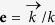, 
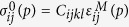
, and 
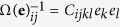
.

The total free energy of the system, *F*, consists of all the above contributions, i.e.,


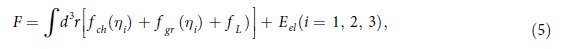


and its variational derivatives with respect to the order parameter fields form the thermodynamic driving force for microstructure evolution during the martensitic or strain glass transitions characterized by the spatial-temporal evolution of the order parameters, following the stochastic time-dependent Ginsburg-Landau equation:


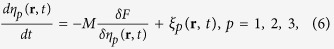


where *ξ* is the Langevin noise term describing thermal fluctuations and *M* is the kinetic coefficient characterizing the rate of the transitions.

Without considering the LFE, the above phase field model has shown that self-accommodation among different martensitic variants during nucleation and growth dominated by the long-range elastic interactions leads to normal sharp martensitic transformations producing long-range ordered (LRO) polytwin domain structures^16^,^28^,^32^,^39^,^40^,^41^ via autocatalysis and collective nucleation. As will be shown below that the presence of the LFE will screen the long-range interactions, suppress autocatalysis and limit the extent of growth, and convert a sharp MT into a smooth STGT, to which the Elinvar anomaly could be attributed. Note that the random temerpature effect^42^,^43^ due also to spatial non-uniformity of point defects could also induce a strain glass state with a continuous transition and, thus, lead to the Elinvar anomaly. Considering the fact that the combination of the GTTE and LFE allows for predictions of all the transition characteristics (including strain glass transition) and the strain-state phase diagrams^32^, which agree well with experimental observations^21^,^32^,^44^, the random temperature effect is ignored for simplicity.

The dynamic storage modulus of the system is calculated through


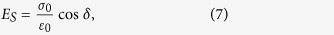


with *ε*_0_ and δ being obtained by fitting the total strain (elastic + inelastic (SFTS)) data from the simulations to equation *ε *= *ε*_*0*_ sin(*tω* – *δ*) with an applied external load given by *σ *= σ_0_sin(*tω*), where *ω *= *2πf* ( *f* is the frequency of strain oscillation), *t* is time, and *δ* is the phase lag between stress and strain. The elastic modulus (i.e., the static elastic modulus) is calculated through fitting the storage modulus data to the Havriliak–Negami equation^45^,^46^, an empirical modification of the Debye equation, which describes the relaxation processes of all ferroic glasses such as cluster-spin glasses, relaxors and strain glasses:


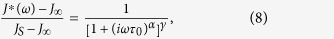


where *J*_s_ and *J*_∞_ are the static compliance and the compliance at infinite frequency, respectively, *α* and *γ* are constants, and *τ*_0_ is the characteristic relaxation time. J*(*ω*) is the frequency-dependent complex compliance,


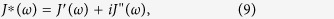


where *J*'(*ω*) and *J*"(*ω*) are the storage and loss compliances, respectively. For the convenience of fitting the storage modulus, Eq. (8) is usually expressed as:


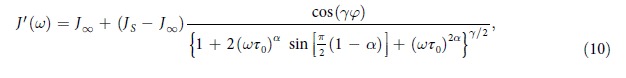


and


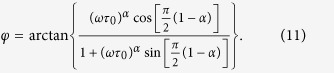


The following parameters in dimensionless units are used in our simulations: the Landau expansion coefficients: 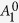 = 0.027, *A*_2_ = 32.0625, *A*_3_ = 33.075; the elastic constants C_11_=115, C_12_ = 67.5, and C_44 _= 58.5; the gradient energy coefficient β = 0.03, and the kinetic coefficient M = 1.0. All the energy terms in the above equations are normalized by a typical chemical driving force for the martensitic transformation, Δ*f*_*A→M*_ = −1.57 × 10^8^ J/m^3^. If we assume the interface energy between A and M to be *γ* ≈ 100 *mJ*/*m*^2^, then the numerical grid size becomes *l*_0_ ≈ 1.0 nm. The temperature dependence of the free energy of A and M phases is determined according to reference^30^. The system size considered in the simulations is 64 × 64 × 64 and periodical boundary conditions are applied along all three dimensions. The real defect concentration in mole fraction, *X,* can be calculated from the dimensionless concentration c according to 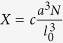
16, where *a* is the lattice constant of the austenite, *l*_0_ is the grid size of the numerical simulations, and N is the number of defect-carrying unit cells in one volume element of the simulation super-cell.

## Results

**Figure 1:**
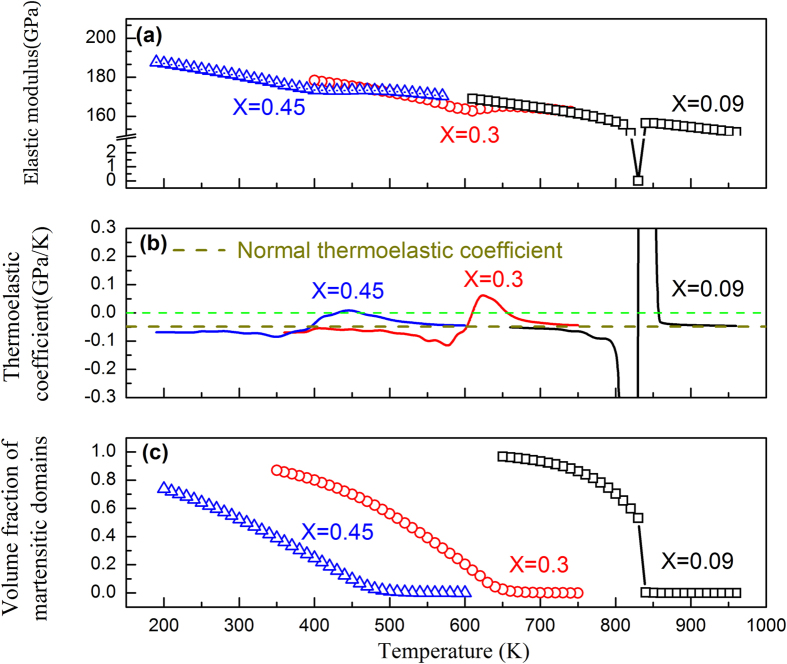
Variations of (**a**) elastic modulus, (**b**) the corresponding thermoelastic coefficient, and (**c**) volume fraction of martensitic domains as function of temperature in doped ferroelastic systems having different point defect concentrations. The open triangles indicate invariance of the elastic modulus over a temperature range of ~100 K.

The calculated temperature-dependence of the elastic modulus (including normal modulus hardening caused by anharmonic atomic vibration and softening caused by phase transition) with different defect concentrations are shown in Fig.1. A normal modulus hardening with a constant thermoelastic coefficient of −0.0484 GPa/K is assumed. At low defect concentration (e.g., X = 0.09), the simulation result (open squares) shows a sharp decrease in the elastic modulus at the transition temperature and then a steady increase after the transition. The corresponding thermoelastic coefficient (Fig. 1(b)) (i.e., the derivative of the modulus with respect to temperature) shows a narrow and sharp peak. As the defect concentration increases (e.g., X = 0.30), the sharp thermoelastic coefficient peak changes into a broadly smeared peak. When the defect concentration reaches X = 0.45, the elastic modulus is almost invariant (open triangles in Fig. 1(a)) and the thermoelastic coefficient (Fig. 1(b)) is nearly zero within a wide temperature range (~100 K). Fig.1(c) shows the change in volume fraction of the M phase, which becomes smoother and smoother as the defect concentration increases. These results suggest that impurity doping in ferroelastic systems is an effective way to adjust MT characteristics and tailor the thermoelastic coefficients.

**Figure 2:**
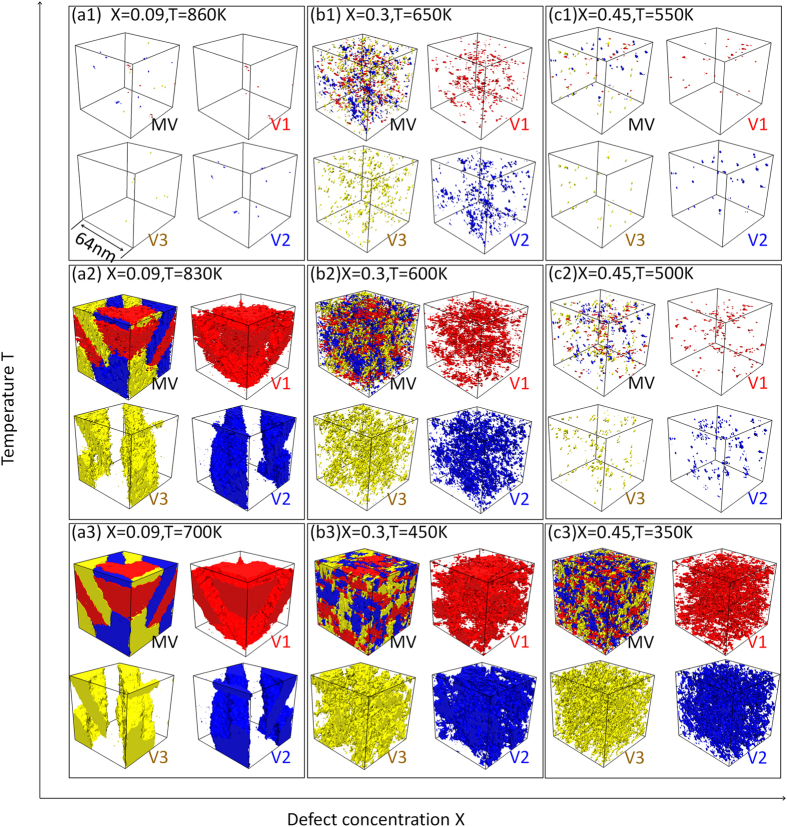
Microstructure evolution in different ferroelastic systems upon cooling. (a1–a3) X = 0.09, (b1–b3) X = 0.3, (c1–c3) X = 0.45. Red, blue and yellow represent the three correspondance variants of martensite. MV indicates micostructures consisting of all three variants, while V1, V2 and V3 represent the individual M variants.

To understand the transition in the characteristics of the MT, microstructural evolution during the MT at different temperatures in three systems of typical defect concentrations (i.e., X = 0.09, 0.30 and 0.45) are shown in Fig. 2. In the case of X = 0.09, the system shows a sudden formation of a long range ordered, self-accommodating and internally twinned multi-variant domain structure (i.e., normal MT) due to autocatalysis. For X = 0.3, the system undergoes continuous nucleation and growth of martensite upon cooling with the formation of relatively large martensitic domains. When the defect concentration reaches X = 0.45, however, the system shows continued formation and confined growth of nanodomains of M upon cooling, which is accompanied by a nearly invariant value of the elastic modulus and a gradual increase in the volume fraction of M over a wide temperature range (as shown in Fig. 1).

**Figure 3:**
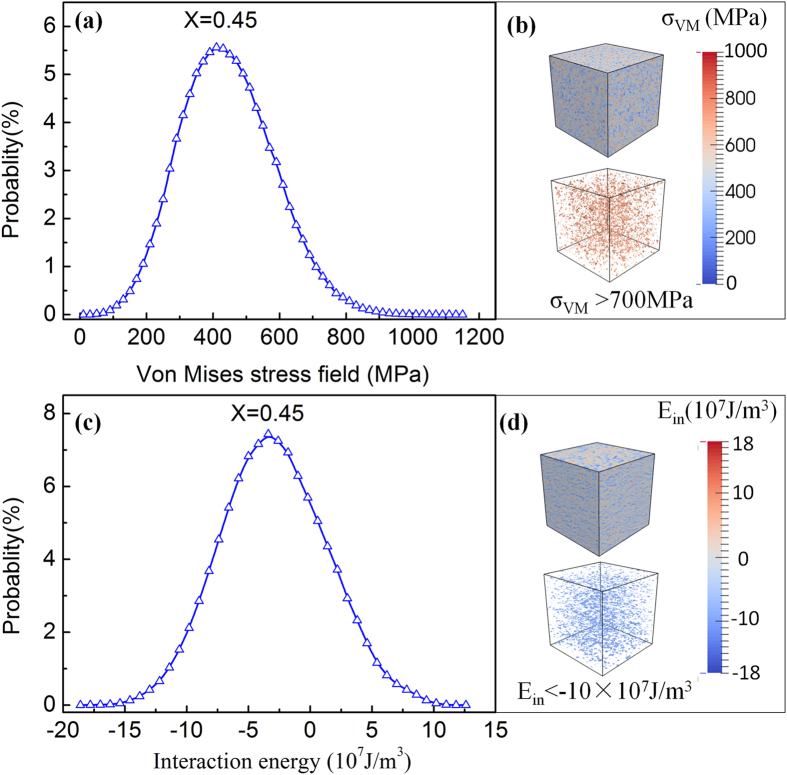
(**a**) Distribution probability of local von Mises stress values generated by the point defects; (**b**) spatial distribution of local von Mises stress field σ_VM_ and the region with σ_VM_ > 700 Mpa ; (**c**) Distribution probability of the values of elastic interaction energy between the point defects and martensitic variant 1; and (**d**) spatial distribution of the elastic interaction energy E_in_ and the region with E_in_ > −10×10^7^ J/m^3^.

To explore the physical nature of the point defects in the Elinvar system (X = 0.45), we calculate the distribution of local von Mises stress values, σ_VM_, generated by the point defects through counting the frequencies of σ_VM_ values within different stress bins in the whole system. The results show a continuous and wide distribution as shown in Fig. 3(a). In Fig. 3(b), we show the corresponding spatial distribution of σ_VM_ (top figure) as well as regions with σ_VM_ > 700 MPa (bottom figure). These results indicate clearly the magnitude and spatial extent of the random stress field generated by the point defects. As a measure of the potency of local stresses on inducing the MT, we calculate the elastic interaction energy (E_in_) between the point defects and a single martensitic variant. The distribution of the interaction energy values, i.e., the potency distribution caused by the point defects, is shown in Fig. 3(c) and the corresponding spatial distribution of E_in_ and the regions of negative interaction energy are shown in Fig. 3(d) by the top and bottom figures, respectively. One can readily see that the interaction energies with negative values are densely populated in the system, with a more or less random spatial distribution. Such random stress and interaction energy fields prefer the nucleation of martensitic nanodomains and prevent the formation of long range ordered internally twinned M domains. As a consequence, only nanodomains of individual martensitic variants are formed.

**Figure 4:**
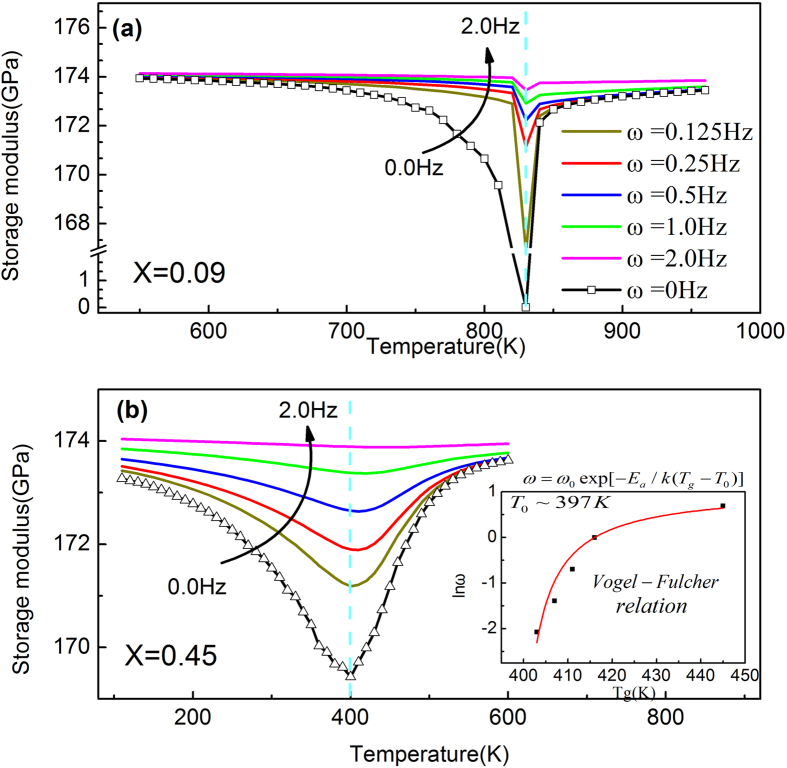
Dynamic storage modulus (without considering elastic modulus hardening by decreasing temperature) at frequency *ω* = 0.125, 0.25, 0.5, 1.0, 2.0 Hz and *ω* = 0 Hz (i.e., static elastic modulus) at defect concentration (**a**) X = 0.09 and (**b**) X = 0.45. Inset describes the Vogel-Fulcher relation between frequency and Tg (i.e., the dip temperature). Dash lines describe the dip position at 0 Hz.

Without considering the normal modulus hardening caused by anharmonic atomic vibration, the corresponding frequency dependence of the storage modulus (i.e., the dynamic elastic modulus caused by the phase transition) of the doped ferroelastic systems (X = 0.09 and 0.45) upon cooling are shown in Fig. 4. For the case of X = 0.09 (Fig. 4(a)), the MT starting temperature (M_s_) does not change with frequency, which agrees with the characteristics of normal MTs^19^,^32^. However, for systems with higher defect concentrations (i.e., X = 0.45) (Fig. 4(b)), the elastic modulus shows an obvious frequency dispersion and the valley temperature (i.e., T_g_) follows the Vogel-Fulcher relationship (as shown in the inset). This is a clear indication of glassy strain freezing in the case of high defect concentration. Note that the static elastic moduli (at 0 Hz frequency) in Fig. 4 are calculated by fitting the simulation data according to the Havriliak–Negami equation.

**Figure 5:**
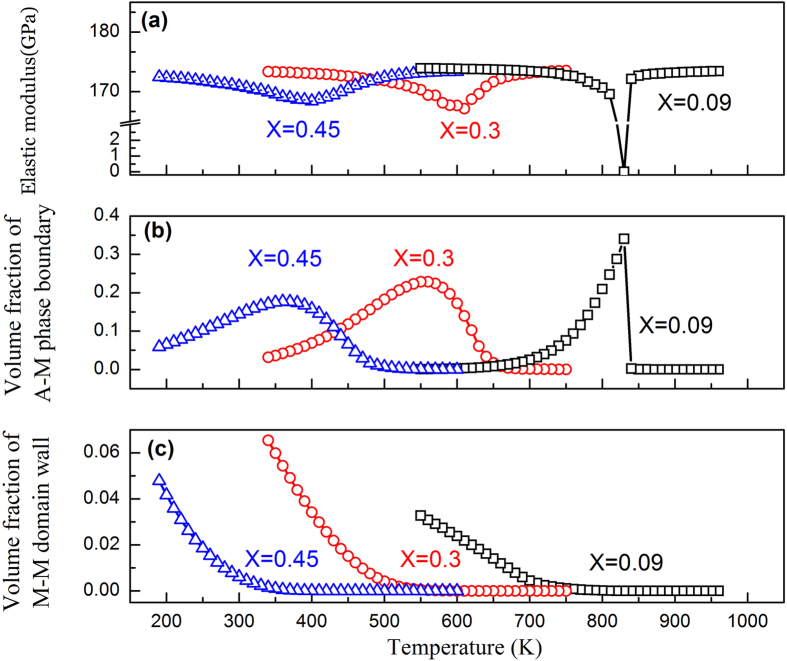
(**a**) Elastic modulus (associated only with the phase transformation), (**b**) volume fraction of austenite-martensite phase boundary, and (**c**) volume fraction of the martensite-martensite domain wall as function of temperature at defect concentration X = 0.09, 0.3, 0.45, respectively.

Further analysis shows that the elastic modulus softening occurs in two steps upon cooling and correlates well with the changes in the fraction of volume occupied by the austenite-martensite (A-M) phase boundaries. The volume fractions of A-M phase boundary and M-M domain wall as functions of temperature in systems having different defect concentrations are shown in Fig. 5(b,c), respectively, together with the corresponding modulus change (Fig. 5(a)). The system with X = 0.09 shows a smooth decrease of the elastic modulus before the formation of any A-M phase boundaries, which should be attributed to the intrinsic modulus softening of the austenite when it approaches to the M_s_ temperature. Subsequently, a much sharper decrease in the elastic modulus is observed when the A-M phase boundary begins to form, which suggests that the increase of the A-M phase boundary during the MT correlates directly to the decrease of the elastic modulus. In contrast, the “diffuse” strain glass transitions in systems with higher defect concentrations (e.g., X = 0.30–0.45) are accompanied by a much lower rate of A-M phase boundary formation and, thus, show a gradual modulus softening. Note that the minimum of the elastic modulus corresponds well to the maximum of the A-M boundary fraction. The formation of the A-M phase boundaries and the intrinsic austenite softening should both contribute to the elastic modulus softening that leads to Elinvar, which is indicated by the observation that Elinvar appears before the formation of martensitic nanodomains, as shown in Fig. 1. Furthermore, the calculated M-M domain wall volume fraction (Fig. 5(c)) shows a monotonic increase upon cooling, which is different from that of the A-M phase boundary. Also note that the total volume fraction of the M-M domain wall is about one-order of magnitude smaller than that of the A-M phase boundary.

## Discussions

The origin of temperature dependence of the elastic modulus in solids when phase transition is absent can be attributed to the anharmonic atomic vibration and thermal expansion^2^,^47^. As described by Wachtman’s equation^2^, the elastic modulus varies as T^4^ at sufficiently low temperatures (e.g., far below the Debye temperature) and linearly with T at higher temperatures. Since the Elinvar temperatures in our simulation are higher than 400 K, we have assumed a constant intrinsic thermoelastic coefficient in our calculations.

**Figure 6:**
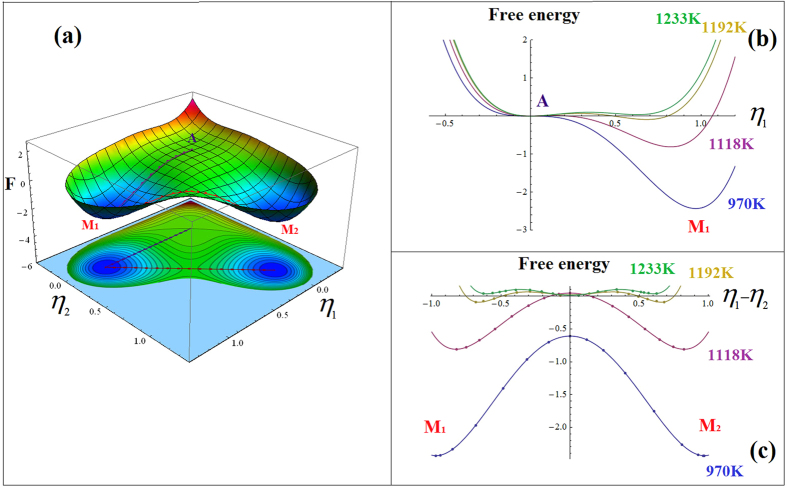
(**a**) Landau free energy surface at 970 K (below martensitic phase transition temperature) with the minimum energy pathways between the austenite (A) and variant 1 of the martensite (M1) and between the two martensitic variants (M1 and M2) indicated, (**b**) Landau free energy curves along the minimum energy pathway between A and M1 at different temperatures, and (**c**) Landau free energy curves along the minimum energy pathway between M1 and M2 at different temperatures.

The Landau free energy given by Eq. 2 describes the local free energy of the system as function of the order parameters that characterize the strain states of different deformation variants of M and are linearly proportional to the transformation strain in proper MTs^28^,^36^,^48^,^49^. The elastic modulus of such a system is proportional to the second derivatives of the Landau free energy with respect to the order parameters^50^. At high temperatures the austenite phase is absolutely stable, i.e., there is only one potential well on the Landau free energy landscape. However, when the temperature approaches to the A → M transition temperature, a second potential well corresponding to the M phase will appear (e.g., when M becomes metastable) (Fig. 6). Upon further cooling, the A phase gradually loses its stability against the M phase, e.g., the potential well corresponding to the A phase becomes shallower and shallower and eventually disappears (i.e., the modulus of the A phase softens to zero), while the potential well corresponding to the M phase becomes deeper and deeper. Thus, within this temperature range the local curvature of the Landau potential at the A phase decreases and that at the M phases increases along the A → M transformation pathway as shown in Fig. 6(b). The transformation pathway is the minimum energy path on the free energy hypersurface determined by using the Nudged Elastic Band (NEB) method^51^ and indicated by the dotted line connecting A and M_1_ in Fig. 6(a).

Furthermore, when both A and M coexist, the center portions of the A-M boundary regions correspond to locations on the Landau potential surface that have negative curvatures (Fig. 6(b)). Similarly, When multiple variants of the M phase coexist, the center portions of the M-M domain wall regions also correspond to locations at the Landau potential surface that have negative curvatures (Fig. 6(c)). Thus nanostructures of mixture of A and different variants of M as seen in a strain glass state could have ultra-low elastic modulus.

For a system having low defect concentration, a single M_s_ is displayed and the intrinsic sharp softening of A and rapid formation of A-M phase boundaries within a narrow temperature range around M_s_ (Fig. 5(b)) lead to a sharp decrease in the elastic modulus of the system, as shown in Fig. 1(a). At lower temperatures when M becomes absolutely stable (i.e., when the A potential well disappears), even though there are M-M domain walls, its volume fraction is small (as compared to the A-M phase boundary, as shown in Fig. 5(b,c)) and thus the intrinsic hardening of the M phase dominates over the softening due to the existence of the M-M domain walls and the overall elastic modulus of the system increases at low temperatures upon cooling.

In contrast, the diffuse strain glass transition in heavily doped ferroelastic systems is characterized by gradual maturation and slow (confined) growth of nanodomains of martensite in a broad temperature range. Because of the effect of random field from point defects, different locations in the austenite will have different potencies to transform into martensite (i.e., embryos of martensite of wide spread of maturities and hence M_s-local_ temperatures) exist in the system. Therefore, nanodomains of martensite start to form at a much higher temperature than the fixed M_s_ in a normal martensitic system but with limited growth because of the confinement from the neighboring domains. This process continues till a much lower temperature than M_s_ accompanying such a smooth transition is a gradual softening of A and gradual formation and evolution of A-M and M-M phase boundaries over a wide temperature range (Fig. 5(b,c)), leading to a much more gradual softening of the overall elastic modulus of the system. When this gradual modulus softening compensates the normal modulus hardening associated with anharmonic atomic vibration, at an appropriate level of defect concentration, the Elinvar anomaly appears.

Even though our simulations are carried out for the classical Elinvar alloy Fe-Ni, with all model parameters chosen specifically for this system, the method used and the mechanism demonstrated are complete general. According to our predictions, other strain glass systems should also exhibit the Elinvar anomaly. To confirm this, we measure experimentally the storage modulus of a different ferroelastic system that has a strain glass transition, Ti_50_Ni_50-x_Fe_x_ (TiNiFe_x_)^21^. Samples of TiNiFe_x_ alloys with x = 2, 4, 6, 8 were prepared by induction melting of high purity metals (>99.9%). All the ingots were capsulated in vacuum quartz tubes, heat treated at 1273 K for 24 h and then quench in water to eliminate compositional non-uniformities. The samples were then cut into 2 × 2 × 60 mm^3^, heated to 1273 K again and held for 1h and water quenched before carrying out the DMA (Dynamic Mechanical Analyzer) test. The storage moduli were obtained by TA Q800 DMA using the single cantilever mode with amplitude of 15 μm (AC field frequency 1 Hz).

**Figure 7:**
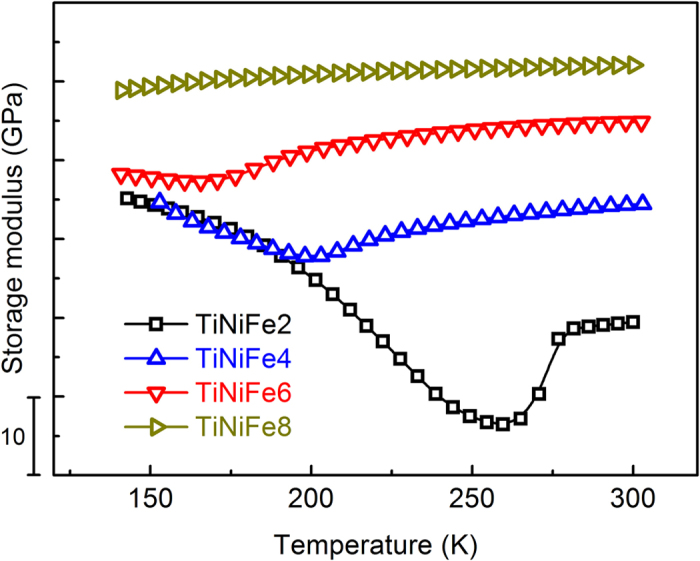
Experimental results of storage modulus in TiNiFeX (i.e., Ti_50_Ni_50-X_Fe_X_, X = 2, 4, 6, 8) systems for different Fe concentration by DMA (Dynamic Mechanical Analyzer).

Figure 7 shows the modulus changes of Ti_50_Ni_50-x_Fe_x_ with different point defect concentrations, in which strain glass transition occurs when x > 6^21^. When the concentration of the dopant, Fe, is low (e.g., x = 2), the system shows a sudden decrease of the storage modulus and the modulus softening becomes weaker as the Fe concentration increases. When x~ 8, the Elinvar anomaly indeed occurs. As a matter of fact, recent experimental studies on, non-magnetic Ti2448 alloys^52^ and Gum metal^20^, which all exhibit the Elinvar anomaly, have confirmed the existence of strain glass transitions in all these systems. Since the strain glass transition takes place at different defect concentration and temperature range in different strain glass systems^21^,^44^, determined by the interplay between the “strength” of the characteristic stress-free transformation strain and the “strength” of the eigenstrain of the defects, the defect concentration and temperature range for the presence of the Elinvar anomaly should also vary among different strain glass systems. This is readily seen from above Elinvar systems mentioned.

## Conclusions

In summary, we have demonstrated a new mechanism to account for low and temperature-independent elastic modulus in both magnetic and non-magnetic Elinvar alloys. Our simulations show that random defects in ferroelastic systems could change a sharp, strong first-order martensitic transformation to a diffuse strain glass transition. Detailed microstructural analysis shows that the abundance of retained austenite and domain boundaries between austenite and martensite in a strain glass state and their evolution during a strain glass transition are responsible for the observed low and temperature-independent elastic modulus. The present study may shed light on exploring new materials having low and temperature-independent elastic modulus through defect engineering and domain boundary engineering in ferroelastic materials.

## Additional Information

**How to cite this article**: Zhang, L. *et al.* A new mechanism for low and temperature-independent elastic modulus. *Sci. Rep.* **5**, 11477; doi: 10.1038/srep11477 (2015).
